# Presidential Address

**Published:** 1906-11

**Authors:** S. H. McKee

**Affiliations:** Americus, Ga.


### PRESIDENTIAL ADDRESS.

By S. H. McKee, D. D. S., Americus, Ga.

Georgia State Dental Society, 1906.

According to a well established custom and the By-Laws of this Society, I am expected, and therefore it becomes my duty as your presiding officer on this the occasion of our Thirty-eight Annual Meeting, to read you an address. I am happy to greet you, and extend to you a most cordial welcome, and earnestly ask your harmonious co-operation, realizing without whic I shall be able to accomplish nothing.

Fresh from the halls of Vanderbilt University seventeen years ago, I made application for membership in your ranks, and you took me in; for fifteen successive years past you have elected me an officer among you. I would do violence to my feelings if I did not on this occasion acknowledge my sincere appreciation of the honors you have conferred, and especially do I thank you for the compliment and honor in the gift of the highest office within your ranks. Whatever of success I may have attained in my profession, I owe it largely to this Society and the inspirations here received.

I acknowledge my appreciation of the valuable assistance rendered by the various committeemen in the preparatory work necessary to this meeting, and especially by Dr. A. M. Jackson, of Macon, Chairman of the Committee on Programs, who has labored earnestly and unceasingly.

I congratulate this Society upon the individual and united pride and interest felt by its members for its present and future welfare, as evidenced by the many letters received during the past year.

Gentlemen, representing a great profession, and yet only a handfull, as it were, but boasting the "very flower" of our profession in this great State, from whose four corners we have gathered ourselves together in this beautiful, historic and far-famed hospitable city of Savannah, we come for social intercourse, mutual benefit and the consideration of such matters as may be of interest to this Society, and tend to upbuild our profession and benefit and protect the people everywhere.

It has not been my purpose to make many suggestions and recommendations for your consideration at this meeting.

It would, perhaps, be more conducive to health to allow some of the magnificent dishes served to you by my predecessors, to digest before feeding you again.

From time to time committees have been appointed to revise the By-Laws of this Society, but only once during a long period has this been done. Seven years have elapsed since the last revision. The committee appointed at the Athens meeting to do this work failed to make a report last year. We have a committee outstanding, which I trust will make a satisfactory report at this meeting.

Last year it was recommended by the Executive Committee that a regular order of business be included in the By-Laws so that the incoming President might have something definite to work upon, and have a regular order of business to carry out. It is not practical for the new President to fully discharge his duties without this.

The illegal practitioner is still loose in Georgia, and the dental parlor man inviteth whom he may devour; a movement is on foot looking towards the organization of a "Dental Protective Association of Georgia;" should this be accomplished, I urge every member of this Society to become a member, as this seems to be the only feasible means of defense. I am in receipt of communications from dentists in different parts of the State, not members of this Society, who are interested in this movement, and pledging their support.

I am satisfied that if such an organization is effected, and the time and place of its annual meetings should be the same as that of The Georgia State Dental Society, our membership will be increased largely by men from said "Protective Association"—dentists coming under the influence of this Society will become members with us.

Since our last meeting, death has invaded our ranks and removed from among us two of our most prominent and beloved co-workers, Drs. W. G. Browne and J. A. Chapple. I would suggest that committees be appointed to prepare suitable resolutions on these deaths, to be passed by this Society, and also, that we hold a memorial service at some time during the meeting, as more expressive of respect, and affording an opportunity to those to make tributes who might feel so inclined.

